# Age-related neuronal loss in the rat brain starts at the end of adolescence

**DOI:** 10.3389/fnana.2012.00045

**Published:** 2012-10-26

**Authors:** Priscilla Morterá, Suzana Herculano-Houzel

**Affiliations:** ^1^Instituto de Ciências Biomédicas, Universidade Federal do Rio de JaneiroRio de Janeiro, Brazil; ^2^Instituto Nacional de Neurociência TranslacionalSão Paulo, Brazil

**Keywords:** aging, number of neurons, brain size, atrophy, neuronal loss

## Abstract

Aging-related changes in the brain have been mostly studied through the comparison of young adult and very old animals. However, aging must be considered a lifelong process of cumulative changes that ultimately become evident at old age. To determine when this process of decline begins, we studied how the cellular composition of the rat brain changes from infancy to adolescence, early adulthood, and old age. Using the isotropic fractionator to determine total numbers of neuronal and non-neuronal cells in different brain areas, we find that a major increase in number of neurons occurs during adolescence, between 1 and 2–3 months of age, followed by a significant trend of widespread and progressive neuronal loss that begins as early as 3 months of age, when neuronal numbers are maximal in all structures, until decreases in numbers of neurons become evident at 12 or 22 months of age. Our findings indicate that age-related decline in the brain begins as soon as the end of adolescence, a novel finding has important clinical and social implications for public health and welfare.

## Introduction

Aging-related losses, and ultimately death, are currently understood as consequences of the progressive damage of living tissues, due to processes that include abnormal carbohydrate metabolism (Davidson, 1979), chromosomal damage (Hastie et al., 1990) and telomere loss (Jaskelioff et al., 2011; Sahin et al., 2011; Heidinger et al., 2012), accumulation of mutations in mitochondrial DNA (Balaban et al., 2005; Wallace, 2005), and molecular damage caused by free radicals produced by the very respiration that keeps tissues alive (Barja et al., 1994; Balaban et al., 2005), with particularly dire consequences for those organs, such as the heart and brain, composed mostly of non-proliferative cells. Indeed, sensorimotor and cognitive decline are characteristic of aging in humans (Schroeder and Salthouse, 2004), although with a somewhat large variation across individuals (Schaie, 1989). The recent increases in human life expectancy coupled to the inevitability of aging make it fundamental to understand the anatomical, cellular, and biochemical processes underlying aging and the causes of the related sensorimotor and cognitive decline.

The age-related deterioration of sensorimotor and cognitive capacities implies that the brain undergoes regressive changes with aging that compromise its function. Indeed, imaging studies of the normally aging human brain point to a tendency toward atrophy (Haug, 1977; Gur et al., 1991; Xu et al., 2000), which has been suggested to result at least in part from loss of neurons (Brody, 1955). Loss of about 15% in subcortical white matter volume in human aging has also been observed (Tang et al., 1997). Further investigations confirmed aging-related loss of neurons in the human neocortex (Coleman and Flood, 1987) and hippocampus (Hp) (Ball, 1977). Those data, however, were later deemed unreliable due to methodological difficulties. In the 1980s, with the advent of unbiased stereology (Gundersen et al., 1988), evidences for age-related neuronal loss were disputed in both humans (West et al., 1994; Pakkenberg and Gundersen, 1997), primates (Gazzaley et al., 1997; Merrill et al., 2000; Keuker et al., 2003) and rodents (Rapp and Gallagher, 1996; Merrill et al., 2001), although confirmed by others (see below).

Up to this day, there is still little consensus on whether cell loss is a key modification associated with brain aging and causally related to cognitive changes. In the rat, in which systematic studies of the cellular composition of the brain are more easily done than in humans, studies have found decreases in numbers of neurons between 3 (young adulthood) and 18 or 24 months of age (middle or old age) in the medial occipital cortex (Diamond et al., 1977), in the visual cortex (Yates et al., 2008), in the basal forebrain, and frontal cortex, which also become particularly vulnerable to neural damage caused by lesions (Wellman and Sengelaub, 1995), in the dorsal prefrontal cortex (Stranahan et al., 2012 but see Yates et al., 2008) and in the thalamus (Diaz et al., 1999). Such cell loss would not be suspected from the size of the different brain structures, given that the rat brain, unlike the aging human brain, continues to grow well into middle age (Sullivan et al., 2006). Other studies, however, found neuronal densities and numbers in the rat brain to be stable over the animal's lifetime, despite varying degrees of cognitive decline, and argued therefore that aged-related cognitive impairment could be a consequence of neuronal dysfunction rather than actual neuronal losses [suprachiasmatic nucleus (Madeira et al., 1995), supraoptic nucleus (Berciano et al., 1995), cerebral cortex (Cx) (Peinado et al., 1997), entorhinal cortex (Merrill et al., 2001), Hp (Rapp and Gallagher, 1996), parahippocampal region (Yates et al., 2008)]

Interindividual variability (Schaie, 1989), which is itself of great scientific interest in the study of aging, may obscure a trend toward neuronal loss and other alterations in normal aging. However, it remains possible that no consensus regarding neuronal cell loss with aging has been achieved because of the possibility of a dissociation between age-related volumetric changes and modifications in numbers of cells in brain tissue, which would obscure the results particularly in those cases where analysis of cell numbers are performed simultaneously with tissue volumetry, or worse, depending on measurements of tissue volume. Modern unbiased stereological techniques (Gundersen et al., 1988) allow the bias introduced by differences in tissue volume to be circumvented, but are still sensitive to a correct choice of experimental design and, most importantly, disector size, which unfortunately too often yields a sampling of an average of less than one neuron per disector (for instance, Rapp et al., 2002).

Here we take advantage of the isotropic fractionator, a direct method for determining numbers of cells that compose brain structures that eliminates structure volume as a confounding variable (Herculano-Houzel and Lent, 2005), to examine how aging affects numbers of brain neurons in the rat. Previously, we used this method to examine the postnatal development of the cellular composition of the rat brain, and found that numbers of neurons change remarkably during the first postnatal month, prior to adolescence (Bandeira et al., 2009). Although in that study we found that pre-adolescent rats had fewer brain neurons than young adults, we did not investigate in detail how numbers of brain neurons change during adolescence [1–2 months; reviewed in (Spear, 2000)] and into young adulthood. The recent finding of cognitive decline beginning at an early, immediately post-adolescent age in humans (Salthouse, 2009) suggests that age-related neuronal loss may start before animals are considered “aged,” and even before cognitive decline is detected (Frick et al., 1995), possibly with great implications for public health. Here we examine how the cellular composition of the rat brain changes with age from pre-adult (1 month) to young, post-adolescent adults (2–5 months) to aged animals (12 and 22 months) by applying the isotropic fractionator to determine the numbers of neuronal and non-neuronal cells found in the Cx, Hp, cerebellum (Cb), olfactory bulb (OB) and remaining brain areas of the animals.

## Materials and methods

### Ethics statement

All animal procedures were certified and approved by the Committee on Ethical Animal Use of the Health Sciences Center (CEUA-CCS), Universidade Federal do Rio de Janeiro, protocol number DAHEICB 010.

### Animals

We examined a total of 38 Wistar rats, of ages 1 month (*n* = 4), 2 months (*n* = 9), 3 months (*n* = 8), 4 months (*n* = 4), 5 months (*n* = 4), 12 months (*n* = 6) or 22 months (*n* = 3). Most animals were males (27 of the 38 animals; 1 month, *n* = 4; 2 months, *n* = 6; 3 months, *n* = 7; 4 months, *n* = 2; 5 months, *n* = 1; 12 months, *n* = 5; 22 months, *n* = 2). All animals were bred in the colonies of the Institute of Biomedical Sciences at the Federal University of Rio de Janeiro, and were checked and treated periodically for pathogens. Animals within age groups 1–5 months were siblings so as to exclude rearing effects as a source of variability within groups. All animals were weaned at 31 days of age, and housed jointly until 2 months of age, then individually from that age on, in standard cages with food and water available *ad libitum*.

Animals were killed by inhalation of ether, weighed and perfused transcardially with 0.9% saline followed by 4% phosphate-buffered paraformaldehyde. Brains were removed from the skull using the foramen magnum as the lower limit, dissected free of meninges and superficial blood vessels, weighed, and post-fixed for 2 weeks or up to 2 months by immersion in 4% phosphate-buffered paraformaldehyde. Brains were dissected into five regions of interest: OB, Cb, Cx, Hp, and rest of brain (RoB), using consistent anatomical landmarks as criteria for dissection. Cb was dissected by cutting the cerebellar peduncles at the surface of the brainstem. Cx comprised all regions dorsolateral to the olfactory tract, excluding the Hp, and was dissected from each hemisphere by peeling it away from the striatum and other subcortical structures under a stereomicroscope. OB was dissected with a cut through the olfactory tract immediately proximal to the bulb. All other brain tissues were pooled and processed together as RoB. The two hemispheres were counted together.

Total numbers of cells, neurons, and non-neuronal cells were estimated as described previously using the Isotropic Fractionator (Herculano-Houzel and Lent, 2005). Briefly, each region of interest was mechanically dissociated in a saline solution with 1% Triton X-100 and turned into an isotropic suspension of isolated nuclei, kept homogeneous by agitation. The total number of nuclei in suspension—and therefore the total number of cells in the original tissue—was estimated in a hemocytometer by determining the density of nuclei in typically four 10 μ l aliquots stained with the fluorescent DNA marker DAPI (4′-6-diamidino-2-phenylindole dihydrochloride), under a Zeiss fluorescence microscope with a 40× objective. Interaliquot coefficients of variation were typically around 0.10, and always below 0.15, as described originally (Herculano-Houzel and Lent, 2005).

Neuronal nuclei from an aliquot of the suspension were selectively immunolabeled overnight, at room temperature, with mouse monoclonal anti-NeuN antibody (Chemicon, MAB377B clone A60 against murine NeuN) (Mullen et al., 1992) at a dilution of 1:200 in PBS. This antibody recognizes reliably all neuronal cells, and no glial cells, except for Purkinje cells and cerebellar interneurons (Weyer and Schilling, 2003), mitral cells of the olfactory bulb, inferior olivary and dentate nucleus neurons, and dopaminergic neurons of the substantia nigra (Cannon and Greenamyre, 2009), in a variety of vertebrate species (Mullen et al., 1992) and beginning at neuronal differentiation in early fetal development (Sarnat et al., 1998). Since we identify labeled nuclei by visual inspection under the microscope and not by automated methods, we could confirm that all NeuN-labeled nuclei in each sample were indeed of neuronal morphology, and that all nuclei of a particular labeled morphology were labeled in the sample.

**Figure 1:**
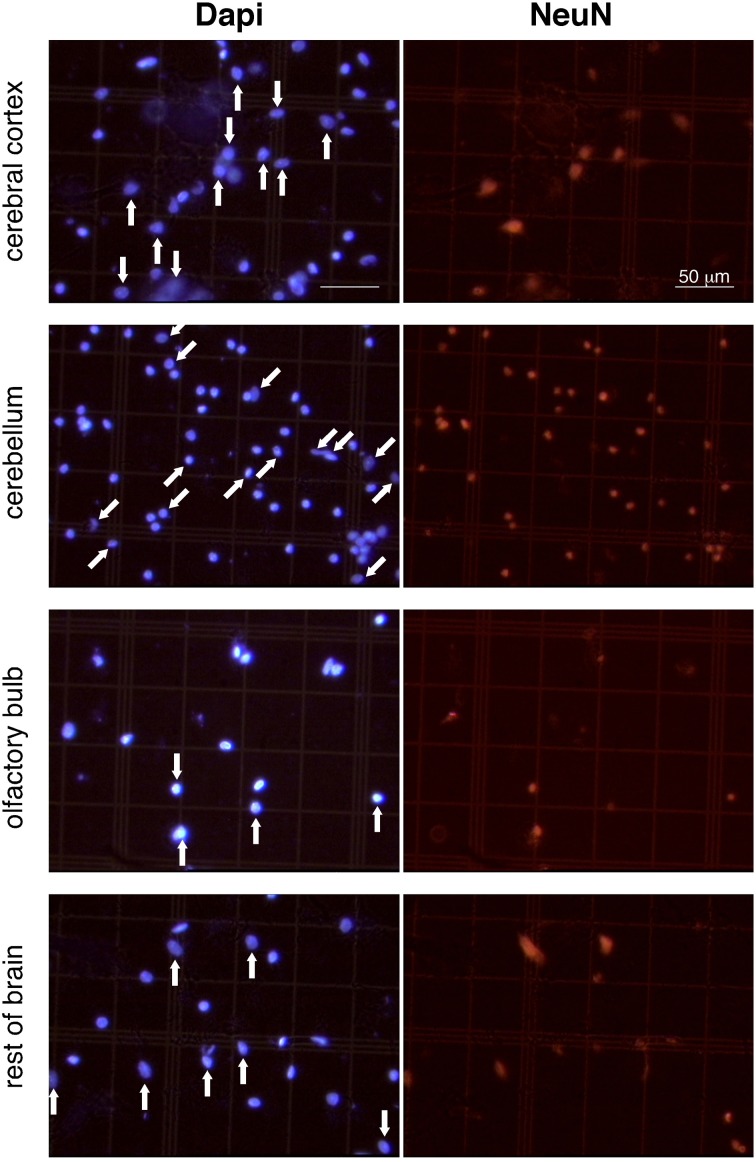
**Appearance of dissociated nuclei and detection of NeuN immunocytochemistry.** Images are representative of the appearance of free cell nuclei of cerebral cortex, cerebellum, olfactory bulb and rest of brain prepared with the isotropic fractionator. Same field is shown on the left (Dapi) and right (NeuN immunocytochemistry). NeuN-positive nuclei are indicated by vertical arrows in cerebral cortex, olfactory bulb, and rest of brain; NeuN-negative labeled nuclei are indicated by oblique arrows in cerebellum (a small group of unlabeled nuclei in the bottom right of the image is not indicated). Notice that isolated nuclei are well separated, intact, and easy to identify; immunocytochemistry is virtually free of background staining; and Dapi-stained nuclei are thus easily scored as NeuN-labeled or unlabeled. All images shown at same magnification. Scale bar, 50 μm.

After washing the nuclei in PBS, they were incubated for 2 h at room temperature with AlexaFluor 555 anti-mouse IgG secondary antibody (Molecular Probes), at a dilution of 1:200 in PBS in the presence of 10% normal goat serum. The neuronal fraction in each sample was estimated by counting NeuN-labeled nuclei in at least 500 DAPI-stained nuclei. NeuN staining is smooth, covers the entire nuclear area is crisp and easily identifiable from the very low background (Figure 1). Controls performed without primary antibody showed no detectable fluorescence in the nuclei. The total number of neurons in each structure was calculated by multiplying the fraction of nuclei expressing NeuN by the total number of nuclei. The number of non-neuronal nuclei was obtained by subtraction.

All statistical analyzes were performed using JMP 9.0 software (SAS Institute, Cary, NC).

## Results

**Figure 2:**
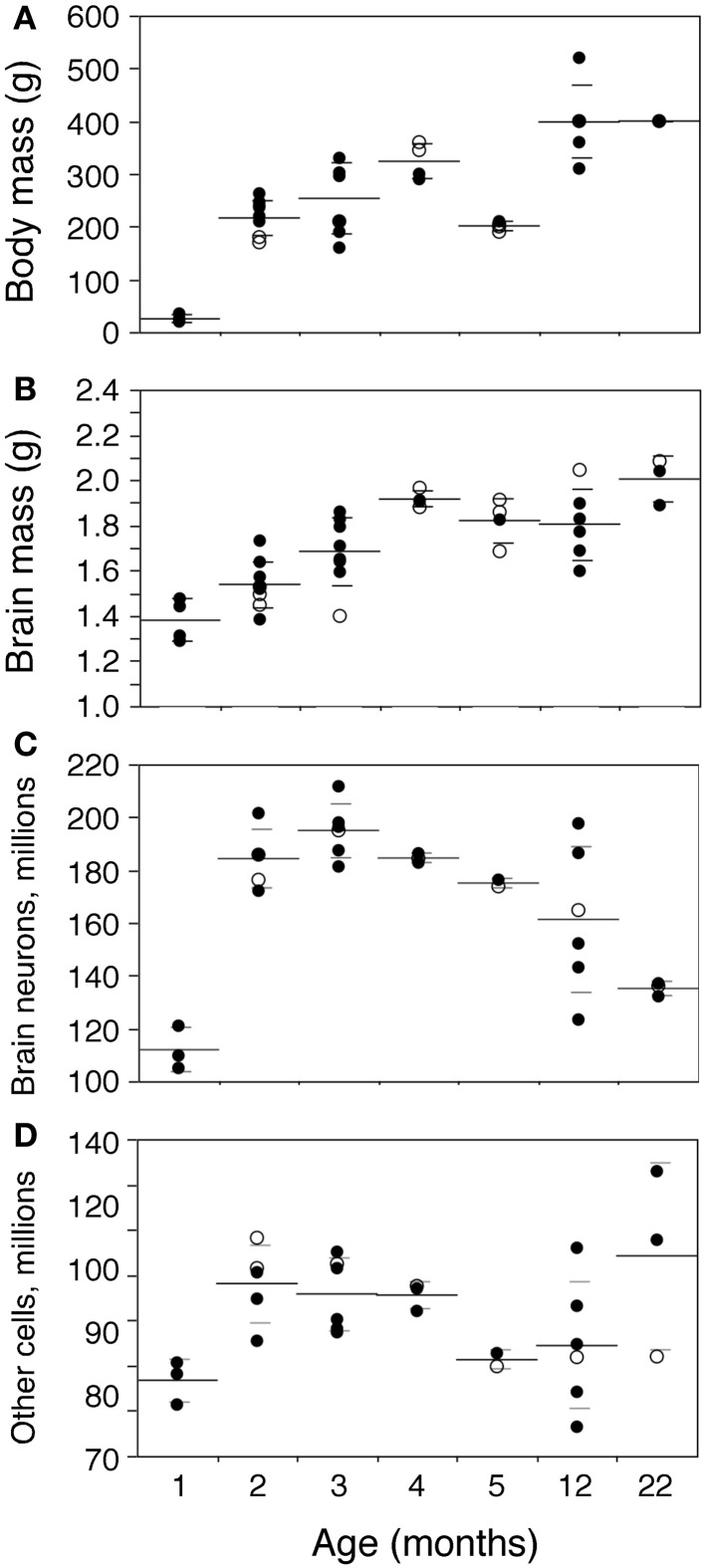
**Changes in body and brain mass with age.** Body **(A)** and brain mass **(B)** increase gradually between ages 1 and 22 months, while total numbers of brain neurons **(C)** increase between ages 1 and 2–3 months then decline progressively with age, and total numbers of other brain cells **(D)** increase between ages 1 and 2–3 months, but then do not change significantly with age. Each point represents one individual. Closed circles, males; open circles, females. Large horizontal line, average value for that age; small horizontal lines, one standard deviation from the mean. “Brain” corresponds to the sum of cerebral cortex, hippocampus, cerebellum, and rest of brain, excluding the olfactory bulb for consistency with other studies reported by our group (Herculano-Houzel et al., 2011). ANOVA shows an effect of age for all parameters (body mass, F-ratio 33.0177, *p* < 0.0001; brain mass, F-ratio 9.0818, *p* < 0.0001; brain neurons, F-ratio 13.6292, *p* < 0.0001; other cells, F-ratio 2.8235, *p* = 0.0356).

**Figure 3:**
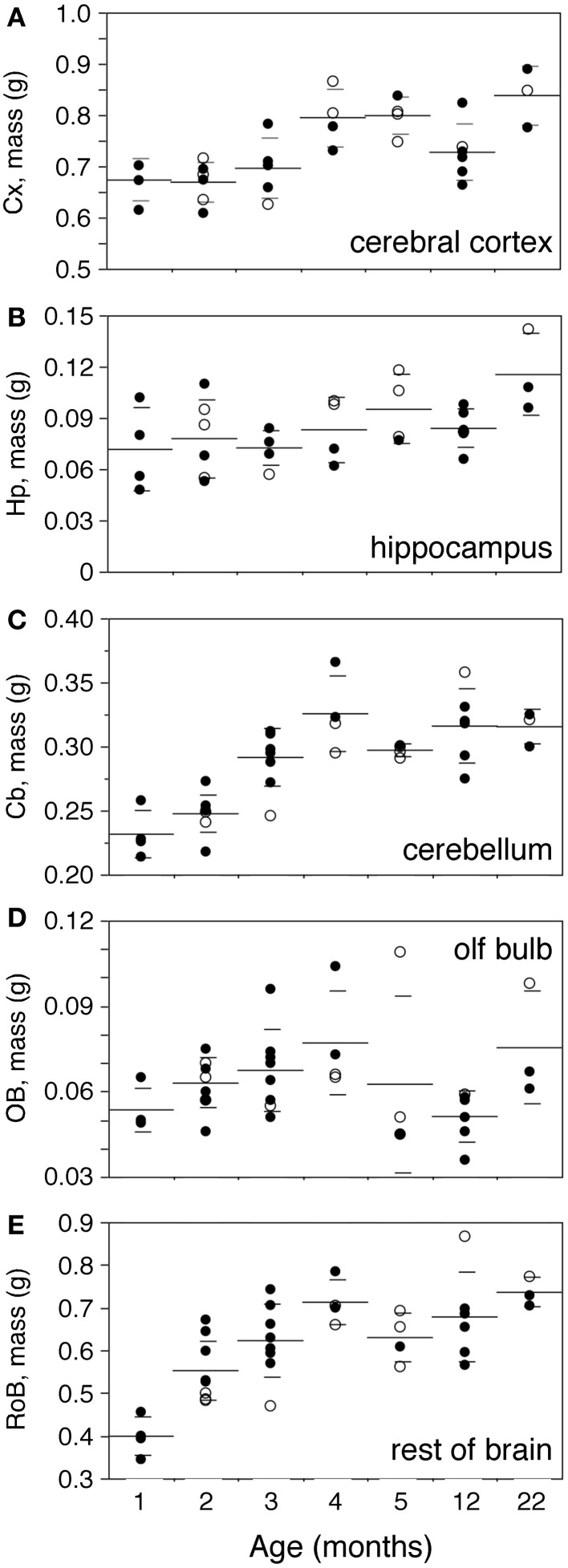
**Progressive increase in brain structure mass with age.** Structure mass increases progressively with age in the cerebral cortex (**A**,Cx), hippocampus (**B**,Hp), cerebellum (**C**,Cb), olfactory bulb (**D**,OB) and rest of brain (**E**,RoB). Each point represents one individual. Closed circles, males; open circles, females. Large horizontal line, average value for that age; small horizontal lines, one standard deviation from the mean. ANOVA shows an effect of age for cerebral cortex (F-ratio 7.4735, *p* = 0.0001), cerebellum (F-ratio 15.1961, *p* < 0.0001) and rest of brain (F-ratio 9.8994, *p* < 0.0001), but not for hippocampus (F-ratio 2.3212, *p* = 0.0642) and olfactory bulb (F-ratio 1.8248, *p* = 0.1266).

Body mass increases 16-fold between 1 and 22 months of age in the rat (Figure 2A). The increase is progressive, with a significant correlation with age (Spearman correlation ρ = 0.7463, *p* < 0.0001). Brain mass also increases in the period, although only by 1.45-fold (Figure 2B; ρ = 0.7188, *p* < 0.0001). A significant progressive increase in mass is observed between 1 and 22 months of age for each of the structures analyzed (Figure 3: Cx, 24% increase in mass, ρ = 0.6217, *p* = 0.0001; Hp, 60% increase, ρ = 0.4193, *p* = 0.0169; Cb, 36% increase, ρ = 0.7877, *p* < 0.0001; RoB, 84% increase, ρ = 0.6894, *p* < 0.0001), except for the OB (Spearman correlation, *p* = 0.9841). No structure shows a tendency toward decreased mass with age.

**Figure 4:**
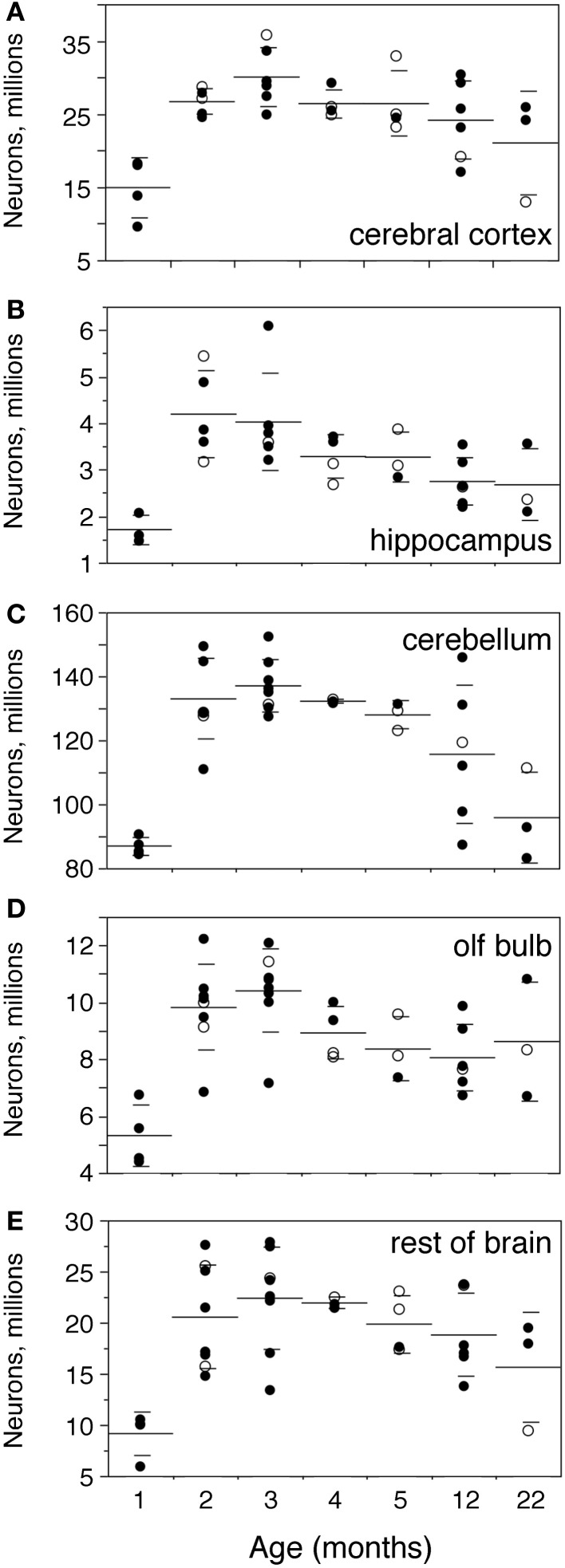
**Numbers of neurons increase until the end of adolescence, then decrease with age.** Numbers of neurons increase between 1 and 2–3 months and then decrease progressively with age in the cerebral cortex (**A**,Cx), hippocampus (**B**,Hp), cerebellum (**C**,Cb), olfactory bulb (**D**,OB) and rest of brain (**E**,RoB). Each point represents one individual. Closed circles, males; open circles, females. Large horizontal line, average value for that age; small horizontal lines, one standard deviation from the mean. ANOVA shows an effect of age for all structures (cerebral cortex, F-ratio 5.8245, *p* = 0.0007; hippocampus, F-ratio 5.2071, *p* = 0.0016; cerebellum, F-ratio 11.1325, *p* < 0.0001; olfactory bulb, F-ratio 7.1240, *p* < 0.0001; rest of brain, F-ratio 5.1337, *p* = 0.0011).

In the same period, all brain structures undergo an initial increase followed by a decrease in their numbers of neurons (ANOVA, all F-ratios >5, *p* < 0.01; Figure 4). Numbers of neurons increase significantly between 1 and 2 months in all brain structures, more than doubling in some (Figure 4; Mann-Whitney, Cx, 78.7% increase, *p* = 0.0143; Hp, 145.0% increase, *p* = 0.0253; Cb, 52.9% increase, *p* = 0.0066; olfactory bulb, 84.8% increase, *p* = 0.0066; RoB, 124.5% increase, *p* = 0.0066). This represents, in the space of 1 month, an addition of 11.8 million neurons to a Cx that initially had 14.9 million neurons; 2.5 million neurons to a Hp that had 1.7 million neurons; 46.0 million neurons to a Cb that had 86.9 million neurons; 4.5 million neurons to an OB that had 5.3 million neurons; and 11.4 million neurons to the RoB, which had 9.1 million neurons at age 1 month (Figure 4). The total number of brain neurons increases 64.7% between 1 and 2 months of age, from 111.9 ± 8.2 to 184.3 ± 11.3 million neurons (Figure 2C).

At 3 months, the total number of brain neurons is even higher compared to 1 month (195.0 million neurons, 74.2% higher than at 1 month, Mann-Whitney *p* = 0.0201), but is not significantly higher from that at age 2 months (*p* = 0.1441). The same pattern is found for each brain structure individually, where numbers of neurons are not significantly different between 2 and 3 months of age (Mann-Whitney, all values of *p* > 0.1), even though all structures (except the Hp) exhibit on average larger numbers of neurons at 3 than at 2 months (Figure 4).

From 3 months on, in contrast, numbers of neurons in each brain structure decrease progressively until 22 months, with a significant negative correlation with age (Spearman correlations: Cx, ρ = −0.5191, *p* = 0.0111; Hp, ρ = −0.6563, *p* = 0.0009; Cb, ρ = −0.7025, *p* = 0.0002; olfactory bulb, ρ = −0.5332, *p* = 0.0073; RoB, ρ = −0.4525, *p* = 0.0264). By 22 months, the Cx and Cb have 30% fewer neurons than at age 3 months (Mann-Whitney, *p* = 0.0389 and *p* = 0.0143 respectively); the Hp and OB have on average 33% and 17% fewer neurons at 22 months than at age 3 months, and although these differences do not reach significance (*p* = 0.0707 and *p* = 0.2207), the two structures have significantly fewer neurons at age 12 months than 3 months (reductions of 32% and 23%, *p* = 0.0131 and 0.0169 respectively). Only in the RoB does the number of neurons not reach significance between age 3 and ages 12 or 22 months (*p* = 0.1962 and *p* = 0.1025 respectively), despite the significant negative correlation between age and number of neurons shown above.

**Figure 5:**
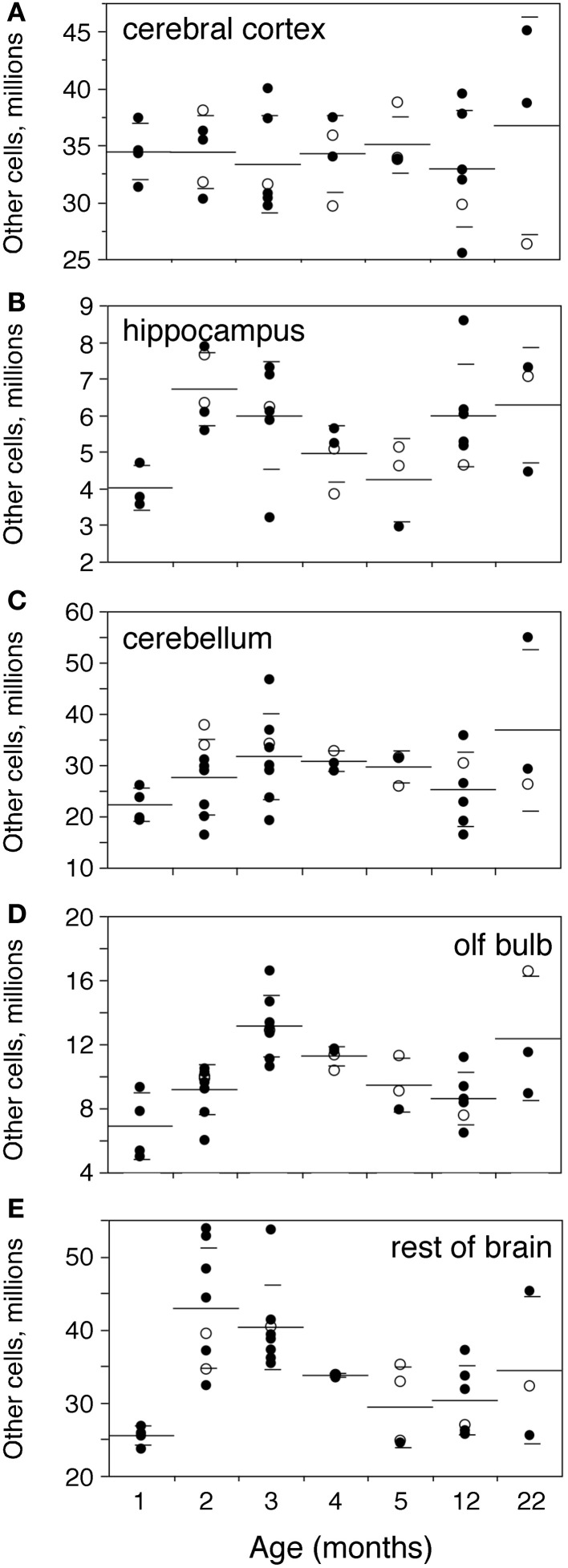
**Numbers of other cells increase until the end of adolescence.** Numbers of other (non-neuronal) cells increase between 1 and 2–3 months and then decrease progressively with age only in the olfactory bulb (**D**,OB) and rest of brain (**E**,RoB), but not in the cerebral cortex (**A**,Cx), hippocampus (**B**,Hp), and cerebellum (**C**,Cb). Each point represents one individual. Closed circles, males; open circles, females. Large horizontal line, average value for that age; small horizontal lines, one standard deviation from the mean. ANOVA shows an effect of age for hippocampus (F-ratio 2.6538, *p* = 0.0418), olfactory bulb (F-ratio 7.2727, *p* < 0.0001) and rest of brain (F-ratio 5.8304, *p* = 0.0004), but not for cerebral cortex (F-ratio 0.2965, *p* = 0.9326) and cerebellum (F-ratio 1.4832, *p* = 0.2200).

Numbers of other, non-neuronal cells are not significantly different between ages 1 and 3 months in the Cx, Hp, and Cb (Mann-Whitney, *p* = 0.4555, 0.1556, and 0.1066, respectively; Figure 5), but are higher by 90.8% in the OB and by 58.1% in the RoB by age 3 months compared to 1 month (Mann-Whitney *p* = 0.0085 in both; Figures 5D,E). As a result, the total number of other cells in the brain is significantly increased by 22.1% at 3 months compared to 1 month (*p* = 0.0282; Figure 2D).

There is no significant difference between numbers of other cells in the brain structures analyzed between 3 and 22 months (Mann-Whitney, all values of *p* > 0.3; Figure 5). However, that might be due to large variance at age 22 months; indeed, again the OB and RoB, but not the other brain structures, exhibit numbers of other cells significantly decreased by 34.5% and 24.8% respectively at age 12 months compared to 3 months (Mann-Whitney, *p* = 0.0055 for both).

Corroborating this decrease in numbers of other cells with age in the OB and RoB, we find a significant trend toward decreased numbers of other cells with age in the two structures between 3 and 22 months (Spearman correlation, ρ = −0.5232, *p* = 0.0087 and ρ = −0.5779, *p* = 0.0031 respectively; Figures 5D,E), but not in the Cx, Hp, and Cb (Spearman correlation, all values of *p* > 0.3; Figures 5A–C).

Inspection of the graphs in Figure 4 shows that there is a fair range of variation in numbers of neurons in each structure in the younger rats, and large variations in numbers of neurons in the older rats, at ages 12 and 22 months. These variations raise the alternate possibilities that (1) in each animal, some structures lose neurons with age while others do not, in which case neuronal loss should not be correlated across structures when all animals are pooled together; and (2) neuronal loss happens in all structures coordinately, but some animals are more prone than others to lose neurons with age, in which case neuronal loss should be correlated across structures when all animals are pooled together.

**Figure 6:**
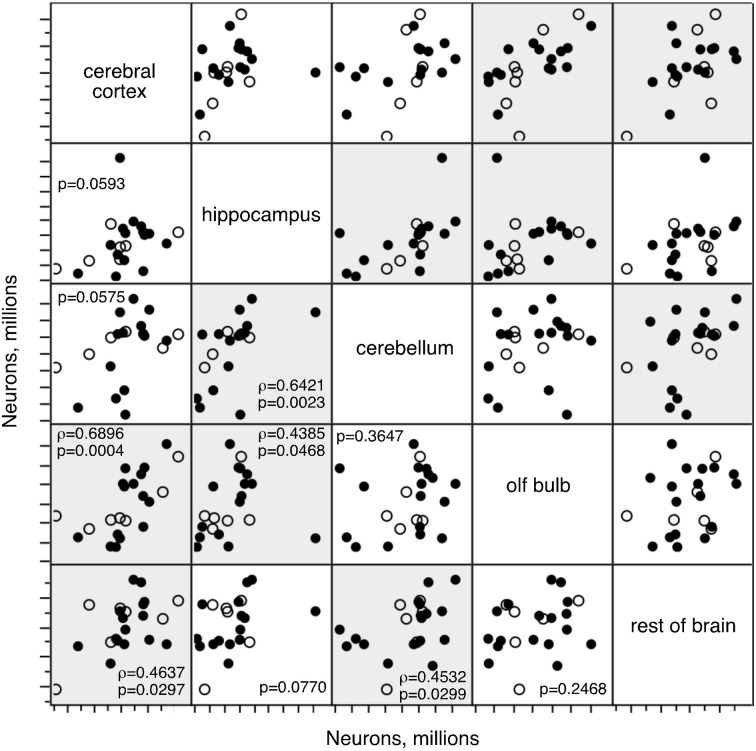
**Numbers of neurons change concertedly across structures.** Correlated variations in numbers of neurons across brain structures in rats aged between 3 and 22 months. Each point represents one individual. Closed circles, males; open circles, females. Spearman correlation *p*-values are listed; where *p* < 0.05, the correlation coefficient is also given. Significant correlations are highlighted.

To distinguish between these alternatives, we performed maximal likelihood factor analysis on the numbers of neurons in each brain structure between 3 and 22 months. We find that while the null hypothesis of no common factor accounting for the variations in numbers of other cells across the structures cannot be safely rejected (*p* = 0.0528), for variations in numbers of neurons the null hypothesis can be rejected at *p* < 0.0001. Two factors account for 60.5% of the variance: factor 1 (rotated loading: Cx, 0.959; olfactory bulb, 0.638; RoB, 0.409) accounts for 31.5% of the variance, and factor 2 (rotated loading: Cb, 0.769, Hp, 0.751, RoB, 0.447) accounts for 29.0% of the variance. These factors are well aligned with the strongest correlations that we find in how numbers of neurons vary between 3 and 22 months across the structures (Figure 6): variations in numbers of neurons in the Cx are correlated with variations in the OB (ρ = 0.6896, *p* = 0.0004) and RoB (ρ =0.4637, *p* = 0.0297), and variations in the Cb are correlated with variations in the Hp (ρ = 0.6421, *p* = 0.0023) and also RoB (ρ = 0.4532, *p* = 0.0299). Together with the factor analysis, these correlations suggest that numbers of brain neurons do not necessarily decrease with age in all animals, but when they do occur in an animal, they tend to happen coordinately across structures. Indeed, across the six animals aged 12 months, we find one animal (rat 49) always among the two animals with the smallest numbers of neurons in the five brain structures; another animal (rat 34) appears among the three animals with the smallest numbers of neurons in three out of five structures; and two other animals (rats 48 and 50) appear among the four animals with the smallest numbers of neurons in four out of five structures (not shown).

## Discussion

Here we show that the adult number of neurons in the Cx, Hp, Cb, OB, and RoB is not yet reached in the 1 month old rat, which is just entering adolescence (Spear, 2000); that these numbers peak at 2–3 months of age, by the end of adolescence (Spear, 2000); and already from that time point, numbers of neurons start to decline progressively with age, until significant losses of 20–30% become apparent throughout brain structures at ages 12 or 22 months. Numbers of non-neuronal cells, on the other hand, only decrease significantly with age, also by 20–30%, in the OB and RoB. The age-related decline in numbers of neurons throughout brain structures and in numbers of non-neuronal cells in the OB and RoB would not be suspected from the steady increase in brain mass with age in the same animals, as observed previously (Sullivan et al., 2006). Although the animals were treated and checked for pathogens, because of the fact that they were not kept in a pathogen-free colony it cannot be ruled out that the age-related neuronal loss observed here may have been not a direct consequence of aging, but a consequence of an age-related vulnerability to other causes of neuronal death, such as stress or diseases (Gallagher et al., 1996). However, because it is unreasonable to expect individuals outside of controlled laboratory conditions to age in perfectly pathogen-free conditions, the age-related decline in numbers of neurons observed here is most likely relevant for the understanding of aging in natural populations.

We cannot at this time address the issue of sex-specific age-related changes in the cellular composition of the brain. It must be noted that males were the majority of the animals analyzed in the present study in all age groups, except 4 and 5 months of age, and therefore, it remains possible that these results apply to males only. However, given that the main effects are found across the other age groups (1 × 3 months, 3 × 12 and 22 months), it is therefore unlikely that the differences observed across age groups were attributable to an uneven distribution of males and females across these groups.

The age-related decrease in numbers of neurons, here gauged from the numbers of NeuN-positive cell nuclei in brain structures, can be safely interpreted as a loss of neuronal cells rather than a loss of NeuN expression given that no coordinate increases in numbers of NeuN-negative (“other”) cells are observed simultaneously. Similarly, the increase in numbers of neurons between 1 and 2–3 months must be attributed to addition of new neurons to the brain structures, rather to a conversion of preexisting NeuN-negative to NeuN-positive cells in the period, because numbers of NeuN-negative are either unchanging (in the Cx, Hp, and Cb) or also increasing (in the OB and RoB) during the period. Importantly, an independent group has just found, using stereology, that total numbers of granule cell neurons increase markedly between 2 weeks and 2 months of age in the mouse OB (by 40%) and dentate gyrus (by 25%) (Cushman et al., 2012). Remarkably, the selective ablation of postnatally generated neurons in the two structures in that study prevented the increase in numbers of neurons until 2 months of age, but did not cause a significant reduction in these numbers at 3, 12 or 24 months (Cushman et al., 2012). This finding, together with decreased neurogenesis already between birth and adolescence (Abdallah et al., 2010; Knoth et al., 2010), makes it unlikely that the age-related net neuronal loss reported here (which is also extensive to the Cx, Cb, and RoB) is simply due to an age-related decrease in the replenishment of adult neuronal populations by ongoing neurogenesis. Direct, widespread neuronal cell death is therefore a more likely explanation for our findings.

The fact that brain mass increases in all structures over time despite a decline in numbers of neurons implies that the average size of the surviving cells, neuronal and/or non-neuronal, increases during aging. GFAP-positive astrocytes have been shown to hypertrophy progressively in the Cb (Sabbatini et al., 1999), in the Hp (Geinisman et al., 1978; Björklund et al., 1985) as well as in the subcortical white matter (Sloane et al., 2000) and gray matter (Adams and Jones, 1982). In neurons, an increase in total cell size could also result from compensatory dendritic hypertrophy (dendritic branching) that can accompany aging-related dendritic spine loss (Mervis et al., 1991).

Several authors have described age-related synaptic loss in different brain structures such as the rat frontal cortex (Nakamura et al., 1999), sensory motor cortex (Markus and Petit, 1987), and Hp (Bondareff and Geinisman, 1976; Geinisman, 1979), with an ensuing decrease in NMDA-dependent glutamatergic transmission in rat Cx and Hp with age (Tamaru et al., 1991). Although synaptic loss might occur in the absence of neuronal loss, it would be a necessary consequence of the neuronal loss described here.

Adolescence has been increasingly recognized as a period of brain remodeling, with profound changes in the cortical gray and white matter (Giedd and Rapoport, 2010), cortical connectivity (Jolles et al., 2011), and brain function (Baird and Fugelsang, 2004). Our findings add to this growing body of evidence by showing that the neuronal population of the brain, in all of its main divisions, has not yet reached its adult numbers by the time that adolescence begins, when rats are 1 month old (Spear, 2000). There is a large potential, therefore, for environmental influences to sculpt the adolescent brain as it gains its final complement of neurons.

Just as we interpret the increase in numbers of neurons from 1 to 3 months of age as the continuation of a developmental process, it must be kept in mind that the later neuronal loss shown here might still be part of such a normal developmental process, with no necessary consequences for cognition. However, it is also possible that age-related neuronal loss does indeed contribute to the cognitive decline associated with aging. Whether or not causally related to cognitive decline, here we show that age-related losses in the brain begin much sooner than when individuals are considered “aged,” and already as soon as adolescence ends, well before animals have been reported to exhibit detectable cognitive deficits (Frick et al., 1995), although our finding that age-related neuronal loss occurs throughout the brain starting already at the end of adolescence matches the recent observation that cognitive decline in humans may start as soon as age 20 (Salthouse, 2009). Although we cannot address whether there is a direct correspondence between neuronal and cognitive losses, a delay between the onset of neuronal loss and noticeable cognitive decline is to be expected (Steffener and Stern, 2012) related to cognitive reserve, compensatory processes, subcapacity use of neuronal populations and/or the sensitivity of testing methods. Indeed, a recent study in mice found that while Purkinje cell loss is apparent at 12 months of age and significant at 18 months of age, impairments in Cb-dependent eye-blink conditioning were only apparent at 24 months of age (Woodruff-Pak et al., 2010).

It will be fundamental to determine whether early changes similar to the ones we observed in rats also occur in humans, given the impact that early intervention could have in such a scenario of earlier-than-expected neuronal loss, to slow down or even prevent aging-related neuronal and cognitive loss, particularly given the efficiency of exercise and cognitive activities in aged individuals (Latimer et al., 2011). While human studies are underway, our findings suggest that public health policies might benefit from encouraging early intervention, beginning in early adulthood, as a means of increasing the likelihood of healthy aging, with the preservation of brain neurons and cognitive capacity.

The large variation in age-related neuronal loss that we find across animals, as in humans (Schaie, 1989), suggests that not all individuals age at the same rate. Importantly, given that the animals in our study, belonging to a non-isogenic strain, were all raised in the same conditions, our data point to a genetic basis of individual susceptibility to aging-related decline, although non-genetic stochastic factors cannot be discarded.

Finally, our data raise an important cautionary note regarding the interpretation of brain volumes: the finding of neuronal loss in the absence of brain atrophy shows that brain volume is not a good proxy of numbers of neurons, and thus points to the possibility that similar neuronal losses occur in the aging human brain even when its volume appears healthy.

### Conflict of interest statement

The authors declare that the research was conducted in the absence of any commercial or financial relationships that could be construed as a potential conflict of interest.
